# Interleukin-22 in Graft-Versus-Host Disease after Allogeneic Stem Cell Transplantation

**DOI:** 10.3389/fimmu.2016.00148

**Published:** 2016-04-19

**Authors:** Baptiste Lamarthée, Florent Malard, Philippe Saas, Mohamad Mohty, Béatrice Gaugler

**Affiliations:** ^1^Centre de Recherche Saint Antoine, INSERM UMR 938, Paris, France; ^2^Université Pierre et Marie Curie, Paris, France; ^3^INSERM UMR1098, Besançon, France; ^4^UMR 1098, SFR FED 4234, Université de Bourgogne Franche-Comté, Besançon, France; ^5^UMR 1098, Etablissement Français du Sang Bourgogne Franche-Comté, Besançon, France; ^6^Service d’Hématologie Clinique, Hôpital Saint-Antoine, Paris, France

**Keywords:** interleukin-22, graft-versus-host disease, allogeneic stem cell transplantation

## Abstract

Allogeneic hematopoietic stem cell transplantation (allo-HSCT) is a potential curative treatment for hematologic malignancies and non-malignant diseases. Because of the lower toxicity of reduced intensity conditioning, the number of transplants is in constant increase. However, allo-HSCT is still limited by complications, such as graft-versus-host disease (GVHD), which is associated with important morbidity and mortality. Acute GVHD is an exacerbated inflammatory response that leads to the destruction of healthy host tissues by donor immune cells. Recently, the contribution of innate immunity in GVHD triggering has been investigated by several groups and resulted in the identification of new cellular and molecular effectors involved in GVHD pathogenesis. Interleukin-22 (IL-22) is produced by both immune and adaptive cells and has both protective and inflammatory properties. Its role in GVHD processes has been investigated, and the data suggest that its effect depends on the timing, the target tissue, and the origin of the producing cells (donor/host). In this review, we discuss the role of IL-22 in allo-HSCT and GVHD.

## Introduction

Allogeneic hematopoietic stem cell transplantation (allo-HSCT) is a potential curative treatment for hematologic malignancies and non-malignant diseases. Because of the lower toxicity of reduced intensity conditioning, the number of transplants is in constant increase. However, allo-HSCT is still limited by complications, such as graft-versus-host disease (GVHD), which is associated with important morbidity and mortality. Acute GVHD is an exacerbated inflammatory response that leads to the destruction of healthy host tissues by donor immune cells. Pioneer studies from Ferrara’s group suggested that the acute GVHD physiopathology consists of three majors steps involving both innate and adaptive immune cells: (i) intestinal damage consequent to the conditioning regimen’s triggering of pathogen-associated molecular patterns (PAMPs) and inflammatory cytokine release, which activate antigen-presenting cells (APC), (ii) activation of donor alloreactive T cells, and (iii) proliferation, migration, and destruction of host tissues (1). Since T cells seemed crucial in the pathogenesis process, an important number of studies focused on T cell pathways and associated cytokines. More recently, the contribution of innate immunity in GVHD triggering has been investigated by several groups and resulted in the identification of new cellular and molecular effectors involved in GVHD pathogenesis (2). Interleukin-22 (IL-22) is produced by both innate and adaptive cells and has both protective and inflammatory properties. Its role in the GVHD process has yet to be determined, but most data suggest that its effect depends on the timing, the target tissue, and the origin of the producing cells (donor/host). In this review, we will discuss the new insights on IL-22 and its role in allo-HSCT and GVHD.

## IL-22 at a Glance

Interleukin-22 has been identified as IL-10-related T cell-derived inducible factor (IL-TIF) (3, 4). This cytokine is a member of the IL-10 family, together with IL-19, IL-20, IL-24, IL-26, IL-28, and IL-29. In humans, IL-22 is an ~20-kDa protein composed of 146 amino acids and shares 80.8% sequence homology with the murine protein. The *IL22* human gene is located on chromosome 12q15, close to the *IL26* and *IFN*γ genes (5). Although IL-10 and IL-22 share only 22.8% sequence homology, both cytokines present a similar secondary structure of multiple α-helixes, which is specific of the IL-10 family (6). At the functional level, glycosylation of IL-22 seems to be more crucial for its binding to its receptor than for its spatial conformation (7).

Murine IL-22 production was reported for the first time in a lymphoma cell line after stimulation by IL-9 and also in lymphocytes treated with concanavalin A, independently of IL-9 (3). In humans, IL-22 expression was initially described in anti-CD3 or concavalin A-stimulated T cells (4, 8). In the immune system, it has been shown that only T cells, innate lymphoid cells (ILC), and neutrophils produce IL-22. By contrast, B cells (activated or not), monocytes, macrophages, dendritic cells (DC), or non-hematopoietic cells are unable to secrete IL-22 (9–13).

Among TCR αβ-chain-expressing T cells, CD4^+^ T cell is the main source of IL-22. Initially, it was thought that only Th1 and Th17 produced IL-22 (10). Later, a new T cell population secreting, exclusively, IL-22 has been described in humans and called Th22. These cells are dependent on the AHR transcription factor and express CCR10, which induces skin-homing properties (14, 15). To a lesser extent, IL-22 can be produced not only by IL-17-producing CD8^+^ T cells (Tc17) (16, 17) but also by a CD8^+^ population secreting exclusively IL-22 but not IL-17, called Tc22 (18). As Th17, γδ T cells also secrete IL-22, they express the ROR-γt transcription factor, in addition to IL-23R, CCR6, and pattern recognition receptors (PRR), such as TLR1, TLR2, and Dectin-1. In addition to IL-22, γδ T cells secrete IL-17 and IL-21, when stimulated with IL-23 and IL-1β, independently of TCR activation (19–21). Similar to what is found in Th17 cells, the transcription factor AHR is required for IL-22 expression but not for IL-17 in the latter cells. In humans, a specific subset of γδ T cells that secrete only IL-22 (Tγδ22) has been recently described. Its differentiation requires IL-1β, IL-23, and TGF-β stimulation (22).

Interleukin-22 secretion by non-T cells has been reported for the first time in murine models with *Citrobacter rodentium* infection (23). In this model, Rag2^−/−^ mice, characterized by the lack of T cells, produced IL-22 at levels equivalent to those found in wild-type (WT) mice. Using immunochemistry methods, the authors initially attributed IL-22 secretion to CD11c expressing cells and speculated that DC could secrete IL-22. Nevertheless, in *in vitro* experiments using IL-23 stimulation, myeloid cells, such as DC, produced a very limited amount of IL-22. These data indicated that secretion of IL-22 by myeloid cells seems unlikely (24). Interestingly, CD11c expression can be enhanced in other cell types and more particularly in ILCs (25). These cells represent a small fraction of the immune cells in lymphoid organs, in epithelial barriers, and other tissues, but they were described as an important source of IL-22 (25–28). Like B and T cells, ILCs are derived from a common lymphoid progenitor. ILCs form a heterogeneous group of different subsets presenting a profile of cytokine secretion and transcription factors similar to that of helper T cell subsets. Spits and Cupedo’s review notably describes functions and phenotypes of these populations (29). Among these heterogeneous populations, only group 3 ILCs produce IL-22. These cells are dependent on GATA-3 and ROR-γt transcription factors for their development and cytokine production, respectively (30).

## IL-22 Target Tissues and Cells

The IL-22 receptor is a heterodimeric protein composed of IL-22R1 and IL-10R2 (4). Since IL-10R2 is ubiquitously expressed, only IL-22R1 expression conditions cellular sensitivity to IL-22. Expression of the latter is mainly observed not only in tissues with a direct interface with the external environment, such as respiratory mucosa (31), gastrointestinal mucosa (32, 33), and skin (13), but also in liver, pancreas, kidney, and thymus (8, 34–36). In these tissues, the responding cells include keratinocytes, dermic fibroblasts, intestinal and bronchial epithelial cells, intestinal subepithelial myofibroblasts, hepatocytes, and acinous pancreatic cells. Importantly, cells from hematopoietic origin, such as monocytes, T and B cells, ILCs, macrophages, and DC, do not express IL-22R1 (10) and, consequently, IL-22 cannot directly activate or inhibit immune cells. In addition to IL-22R1, a soluble receptor for IL-22 called IL-22-binding protein (IL-22BP) is encoded by another independent gene *IL22R2a* (37, 38). IL-22BP is homologous to the extracellular chain of IL-22R1 but is only a secreted protein that does not result from a cleaved membrane protein. It is constitutively expressed in several tissues, such as lymph nodes and intestine (39–41). IL-22BP is secreted by DC expressing CD103 and CD11b in murine intestine (42, 43). In humans, IL-22BP is also expressed by DC and is drastically decreased in the presence of IL-18, or after DC maturation (42, 43). More recently, it has been shown that eosinophils are the most important source of IL-22BP in human healthy gut and contribute to an overproduction of IL-22BP in the inflamed mucosa of inflammatory bowel disease (IBD) patients (44).

## Biological Functions: Between Tissue Regeneration and Inflammation

Interleukin-22 signaling *via* its receptor induces Jak1 and Tyk2 activation, leading to the activation of signal transducer and activator of transcription (STAT) family transcription factors, especially STAT3 (11, 12), but also STAT1 and STAT5 (4, 8, 45, 46). Moreover, mitogen-activated protein kinases (MAPK) pathways, including Erk1/2, JNK, and p38 phosphorylation, are also induced by IL-22 (12, 32, 47, 48). Animal models using IL-22-deficient mice or IL-22 neutralizing antibodies led to the identification of inflammatory or protective roles of IL-22. Interestingly, the IL-22 protective properties are associated with crucial biological functions of STAT3 activation in target cells (49). Indeed, STAT3 induces cell activation, proliferation, and survival *via* anti-apoptotic genes. Thus, IL-22 participates on mucosal homeostasis and epithelial barrier integrity. Murine models suggest that IL-22 plays a major role in intestinal regeneration. For instance, after mechanical wound of the colon, IL-22-deficient mice showed a delayed wound healing as compared to WT animals (46, 50). In dextran sulfate sodium (DSS)-induced or after CD4^+^CD45RB^high^CD25^−^ transfer colitis models, IL-22-deficient mice showed a more important loss of weight and a decreased survival as compared to WT mice (33, 51). Furthermore, in a murine model of IBD, IL-22 can be therapeutic. Indeed, Sugimoto’s group used an interesting microinjection-based gene-delivery approach to directly transfer the *IL22* gene into the large intestine of mice *via* a non-microbial vector and observed a decrease in inflammation, notably by the induction of mucins, which limit microbial flora and host cell interactions (33). In addition, IL-22 induces the secretion of several antimicrobial proteins by epithelial cells such as the regenerating islet-derived proteins Reg3γ and Reg3β, thus limiting infection by extracellular pathogens (52). IL-22 participates in antimicrobial defense by the induction of S100 family proteins, such as S100A7, S100A8, and S100A9, which sequestrate essential elements of bacteria, including iron and zinc. IL-22 also induces β-defensins that are able to destabilize bacterial membranes (11). In skin, *in vitro* tests showed that IL-22 treatment of keratinocytes after a lesion favors cutaneous regeneration (53). In conclusion, IL-22 is essential for epithelial barrier integrity and tissue regeneration after different lesions. Although transgenic mice overexpressing IL-22 presented induction of psoriatic keratinocyte alterations and a high early mortality (54), IL-22 administration to healthy mice does not seem to induce acute or severe inflammation (11, 55). These data suggest the possibility of using this cytokine in human therapy targeting tissue regeneration.

Recently, a phase I clinical trial evaluating IL-22 safety in healthy volunteers has been initiated^1^^,^^2^ (56).

Despite its protective properties, IL-22 is also known to be negatively involved in several inflammatory diseases. In this context, the correlation between the level of IL-22 expression and the severity of the disease has been reported. In an endotoxemia model, IL-22 administration does not inhibit the pro-inflammatory cytokine production, such as TNF-α, IL-6, and IFN-γ, during the early phases of the disease. Moreover, in the late phase, IL-22-deficient mice presented a better survival, showing that IL-22 takes part in the systemic inflammation (57). This notion is also illustrated by another study showing that transgenic mice that artificially express IL-22 receptor on the surface of T cells showed a normal growth initially but presented a multi-organ systemic inflammation after 2 or 3 months of life (58). More specifically, IL-22 participates in the pathogenesis of rheumatoid arthritis both in humans and mice (48, 59). In a murine model of collagen-induced arthritis, the incidence of the disease is decreased in IL-22-deficient mice, associated with a decrease of synovial expression of IL-1β, IL-6, TNF-α, and MMP9 (60). Psoriasis is another disease in which IL-22 is clearly pathogenic and is a key mediator in its late stage, leading to keratinocyte lesions. Thus, a strong expression of IL-22 mRNA is observed in skin lesions from patients as compared to that in healthy skin (61). The serum level of IL-22 is also correlated with disease severity (12). In animal models, IL-22 blockade or IL-22 deficiency is associated with a less severe form of experimental psoriasis (62). Finally, mice that artificially overexpress IL-22 develop psoriasis-like spontaneous lesions (54). The main effects of IL-22 in the skin consist of keratinocyte differentiation and induction of pro-inflammatory effectors, such as CXCL5, IL-20, MMP1, and MMP3 (45, 63, 64). Overall, the effects of IL-22 seem to be protective or pathogenic according to the inflammatory context, the inflammation site, and affected tissue, as well as the cytokines present in the environment.

## IL-22 in the Pathophysiology of Graft-Versus-Host Disease: Increase of Inflammatory Response or Tissue Protection?

Graft-versus-host disease results from activation of donor T cells that recognize and destroy host tissues, such as skin and intestine. Given the IL-22 properties in these tissues, several groups assessed IL-22 contribution in acute GVHD models. We and others used IL-22-deficient grafts and observed that acute GVHD severity was decreased as compared to transplantation with WT grafts (65, 66). These data are in line with the critical role of IL-22 in systemic inflammation. Indeed, in a model where systemic IL-22 administration was performed *via* an adenovirus strategy, it was observed that such exposure o IL-22 induced an acute-phase response that could be detected in the blood and the liver (67). Zhao’s group showed that the main producers of IL-22 involved in the alloreactive immune response of GVHD were donor CD4^+^ T cells that carried CD62L^−^ CD44^high/low^ surface markers, corresponding to effector memory or recently activated T cells (68). Their group also demonstrated that systemic administration of IL-22 exacerbates murine GVHD, confirming the deleterious contribution of IL-22 to this disease (69). Similarly, they showed that IL-22 contributes to effector T cell expansion and Treg decrease. These data are in line with our observations showing that Treg were increased in the spleen and mesenteric lymph nodes of recipient mice transplanted with IL-22-deficient grafts (66). Several experimental reports suggest that Treg can limit alloreactive responses and reduce GVHD severity in mice (70, 71). The expansion of Treg in the absence of IL-22 could be a consequence of reduced inflammation in the GVHD context, rather than an active mechanism.

In a chronic GVHD murine model, Okamoto’s group reported that more than 70% of IL-22-producing cells are CD4^+^ T cells. They treated recipient mice with an anti-IL12p40 mAb and observed a decrease of pathogenic Th1/Th17 cells that secrete IL-22. This decrease was associated with those of GVHD severity and IL-22 levels in the serum of recipient mice (72). In human GVHD, the effect of IL-22 has not yet been fully established, although its expression seems to be increased in both acute and chronic GVHD. In acute GVHD, Brüggen et al. found a clear increase of IL-22 messenger RNA and IL-22-producing CD4^+^ T cells in the skin (73). In active chronic GVHD pediatric patients, a recent report demonstrated an increase of IL-22 expression in peripheral blood mononuclear cells as compared to that in patients with no GVHD (74).

One of the pathogenic mechanisms of donor-derived IL-22 may be due to its synergy with type-I IFN that is produced during the allogeneic response. Recently, we showed that donor-derived IL-22 can synergize with type-I IFN to increase murine GVHD (75). At the protein level, this IL-22/type-I IFN synergy could result from the direct interaction between their relative receptors: the IFNAR physically interacts with the IFN-γ receptor and facilitates phosphorylated STAT1 (PSTAT1) homodimer generation (76). A similar interaction has also been reported between the IFNAR and IL-6 receptors (gp130) (77). It is not yet known whether the IFNAR/IL-22R interaction is physiologically relevant.

Interestingly, intracellular STAT1/STAT3 polarization can be controlled by IL-22 through the SOCS proteins (78). SOCS1 and SOCS2 are responsible for STAT1 inhibition (79). Recently, a report showed that SOCS1 expression was abolished in acute or chronic GVHD patients, suggesting that IL-22-induced STAT1 is no longer inhibited in this context (80). In host cells, it was shown that GVHD induction is associated with a rapid phosphorylation of STAT1 in the spleen and the liver (81). In that model, the use of a histone deacetylase (HDAC) inhibitor to limit murine GVHD led to a local and systemic decrease of STAT1 activation, together with a decrease of pro-inflammatory cytokine production. In chronic GVHD patients, STAT1 activation by type-I IFN seems to be critical in oral mucosal inflammation. Indeed, a higher level of activated STAT1 in keratinocytes has been reported in severe chronic GVHD patients as compared with those patients with no oral lesions. In this context, STAT1 activation is associated with an increase of Th1 inflammation mediators, such as CXCL9 (82). These data are in line with our observation showing that both STAT1 and CXCL10 activation is increased in the colon of gastrointestinal GVHD patients (75).

Given the critical role of pro-inflammatory cytokines in GVHD, signal transduction inhibition seems to be a promising therapeutic approach. Janus kinases (Jak) are intracytoplasmic proteins that trigger cellular signalization leading to cytokine secretion through activation of STAT proteins (83). In preclinical models, Jak2 and Jak3 inhibitors are efficient to limit GVHD (84–87). Our recent results showed that treatment with Jak inhibitors could also limit STAT1 activation and CXCL10 expression in intraepithelial cells (IEC).

There has been recent progress in the knowledge on the effects of CXCL10 family of chemokines in the inflammatory process. CXCL9 and CXCL10 can induce naive T cell differentiation to Th1 in a STAT1-dependent manner. Paradoxically, CXCL11 is known to bind another epitope of CXCR3 and to induce IL-10 producing T cells (88). CXCL10 expression induced by IL-22/type-I IFN synergy and STAT1 activation in host mucosal cells could participate in both Th1 recruiting and Th1-like inflammation maintenance by influencing T cell polarization. Interestingly, we previously showed that plasmacytoid dendritic cells (pDC) able to secrete IFN-α infiltrated both intestinal mucosa and cutaneous tissues in GVHD patients (89, 90). The relationship between IL-22 and pDC is suggested by data showing that IFN-α produced by pDC can increase the expression of IL-22R1, the specific subunit of IL-22R, in keratinocytes (91). Moreover, in humans, the differentiation of Th22, which express CCR10, CCR6, and CCR4 and secrete IL-22 but not IL-17, seems to be pDC dependent (14). Thus, IFN-α and IL-22 may participate in epithelial lesions at the skin or intestinal level, by an amplifying loop to sustain Th1 inflammation.

Despite our observations, we cannot conclude on the negative impact of IL-22 in allo-HSCT context. In addition to donor-derived T cells, other cells could secrete IL-22 during allo-HSCT. For instance, ILCs located in the gut of host mice were shown to secrete large amounts of IL-22 under IL-23 stimulation and could resist the conditioning regimen. In the absence of GVHD reaction, host ILC-derived IL-22 is protective to intestinal stem cells (65). A high level of donor-derived ILCs in the blood of allo-HSCT patients is associated with a less severe GVHD (92) but their capacity to infiltrate GVHD target tissues and their effects remain to be deciphered. A recent report from van den Brink’s group showed that IL-22 can promote ISC-mediated epithelial regeneration. Using *ex vivo* organoid culture, they demonstrated that group 3 ILCs secrete IL-22, which increases the growth of small intestinal organoids. Recombinant IL-22 treatment induced STAT3 activation in Lgr5^+^ ISC and was crucial for IL-22-mediated epithelial regeneration. Moreover, IL-22 treatment *in vivo* also enhanced the recovery of ISC and intestinal regeneration and reduced mortality from GVHD in transplanted mice (93). These observations clearly underline the essential role of IL-22 in damage-induced regulation and maintenance of the ISC compartment.

## IS IL-22 a Potential Therapeutic Target in GVHD?

**Figure 1:**
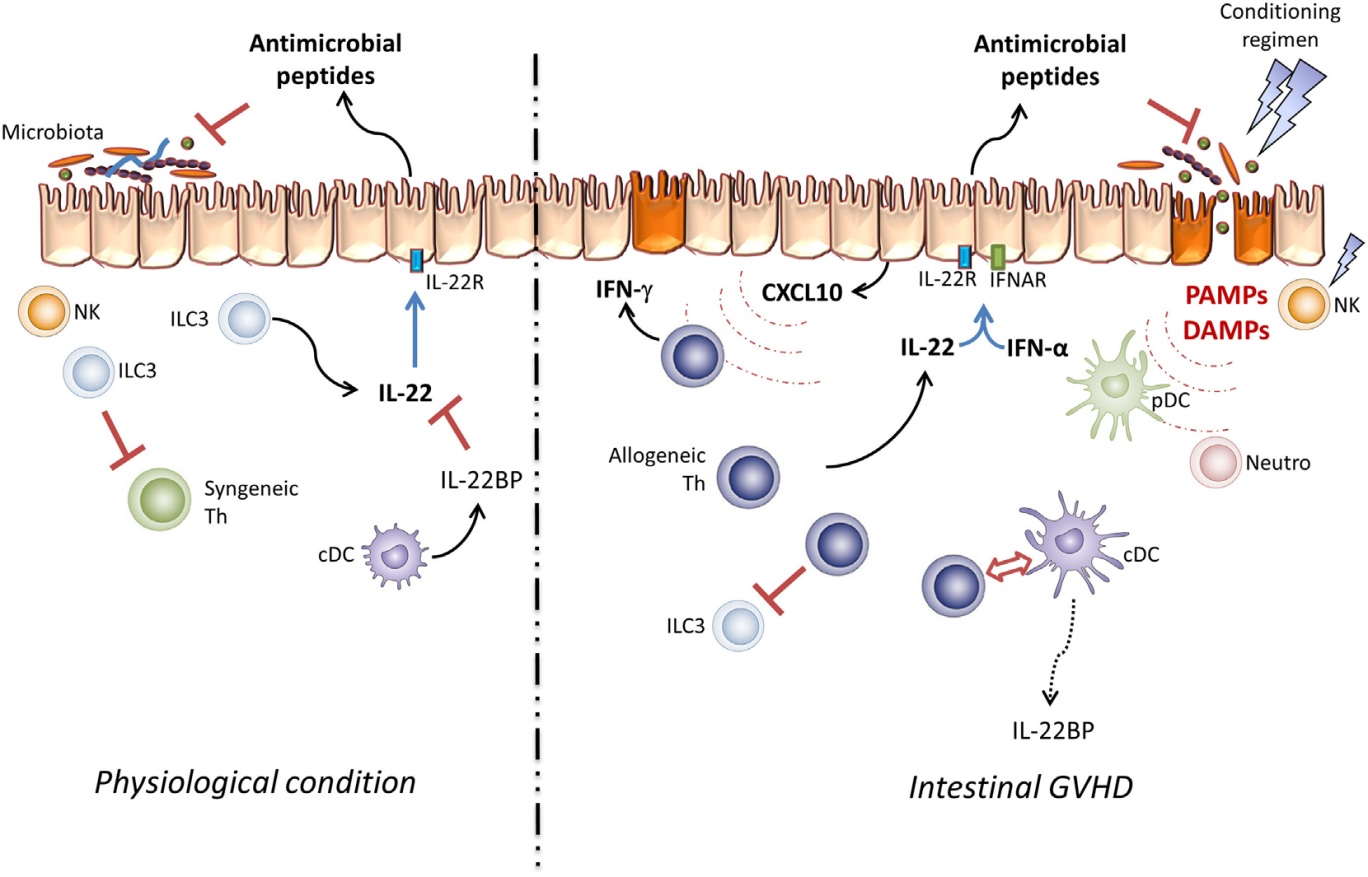
**Pathogenic effect of IL-22 in GVHD**. Under physiological conditions, resident ILC3 cells take part in the intestinal microbiota and mucosal infiltrating lymphocyte homeostasis *via* IL-22 secretion. In this setting, IL-22 activity is controlled by IL-22BP produced by immature DC. In the allo-SCT setting, the conditioning regimen leads to epithelial barrier damage and host NK cell elimination. These lesions increase DAMP and PAMP secretion and induce pDC, neutrophil, and antigen-presenting cell infiltration, processes which activate donor T cells through the “cytokine storm.” Thus, IL-22 and type-I IFN take part in CXCL10 expression and induce Th1 inflammation.

The effect of IL-22 in allo-HSCT seems complex (Figure 1). Thymic RORγt^+^CCR6^+^NKp46^−^ILCs are crucial to secrete IL-22 that contributes to thymic regeneration after total body irradiation in mice (36). Post-transplantation IL-22 neutralization could have a direct impact on thymus regeneration and negatively influence immune reconstitution. The thymus regeneration seems more important during the first 2 weeks post-transplantation after IL-22 treatment, but after the second week, the size of the thymus is comparable to that of non-treated mice (69). However, a recent report showed that IL-22 administration to host mice is associated with a more severe intestinal GVHD. These data suggest that an increase in IL-22-dependent thymus regeneration is not correlated with less GVHD, but that IL-22 could be pathogenic for some tissues and protective for others in the same allogeneic context.

**Figure 2:**
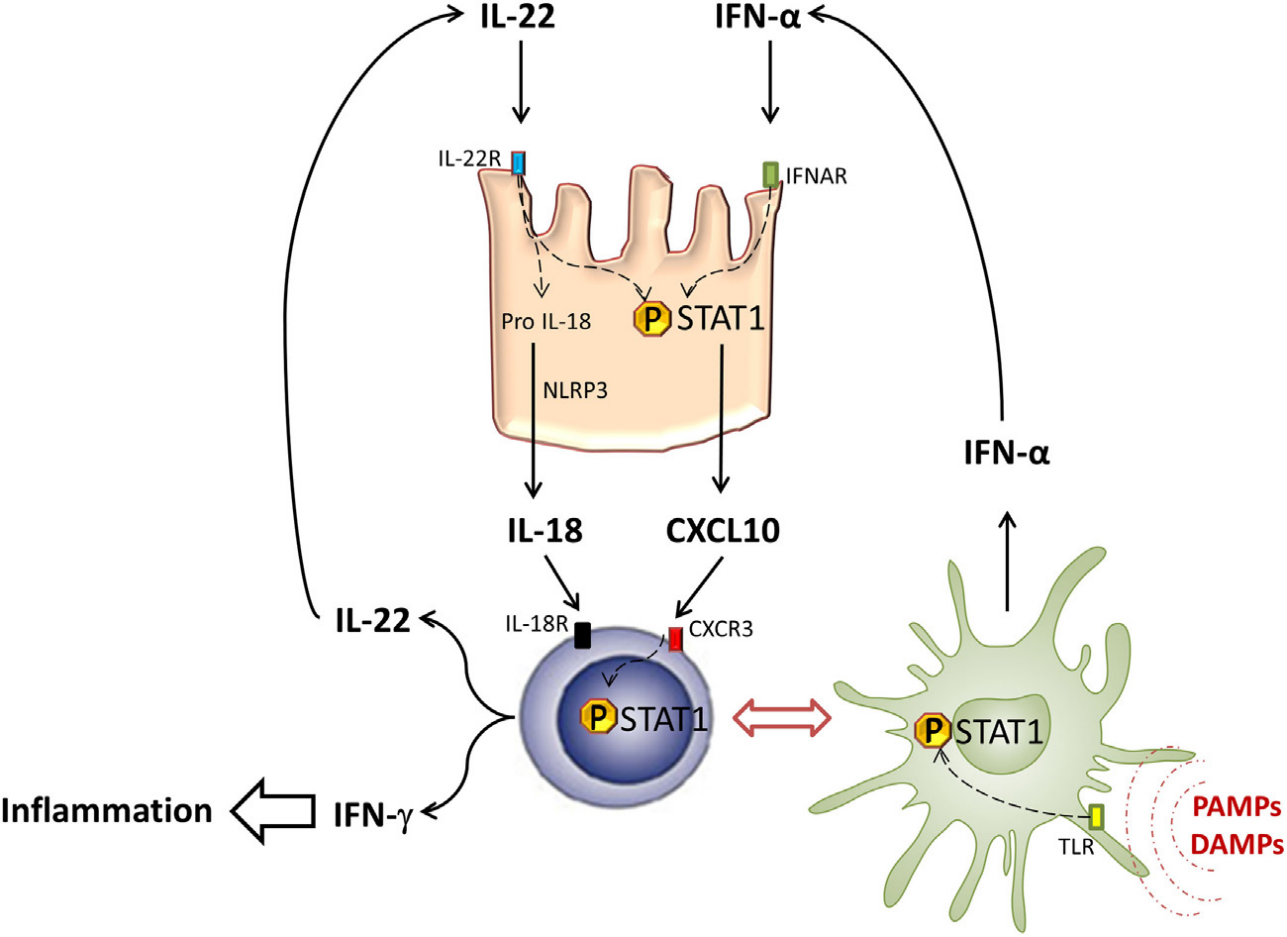
**STAT1 in intestinal inflammation**. In response to PAMPs and DAMPs, pDCs (green cells) infiltrate intestinal tissues and secrete type-I IFN after TLR activation and STAT1 phosphorylation. Type-I IFN induces STAT1 activation in intestinal epithelial cells (pink cells), leading to local CXCL10 expression. CXCL10 induces CXCR3^+^ T cell (blue cells) recruitment and their differentiation into Th1, able to produce type-II IFN under STAT1 dependence. Thus, Th1 cells participate in local inflammation and also secrete IL-22 that contributes to STAT1 activation in IEC.

Paradoxically, donor-derived IL-22 aggravates intestinal damage during GVHD (66). It has been recently reported that IL-22BP expression is maximal when the intestinal barrier is preserved (42). Conversely, tissue lesions and bacterial translocation decrease IL-22BP expression. The mechanism underlying the expression of IL-22BP by DC is inflammasome dependent (94). Thus, IL-18 can inhibit IL-22BP expression by DC (95). In the GVHD context, the contribution of IL-18 remains controversial (96, 97). Nevertheless, Munoz and colleagues recently described an important property of this cytokine. They showed that IL-22 maintains homeostatic IL-18 expression in epithelial cells and IL-8 can be augmented during infection. Moreover, active IL-18 is responsible for a unique inflammatory feedback loop to amplify Th1 cell-mediated immune response (98). Whether donor-derived IL-22 can amplify Th1 response through IL-18 secretion in the GVHD context remains to be addressed (Figure 2). The decrease of IL-22BP expression probably increases bioactive IL-22 at local level. These observations should be confirmed in allo-HSCT patients. Interestingly, antimicrobial peptides, such as Reg3α, that are produced by Paneth cells in response to IL-22 are detected in the blood of GVHD patients and have been validated as intestinal GVHD biomarkers (99). These data are in line with Eriguchi’s group observations, which showed an increase of defensin expression in GVHD mice, which confirm that IL-22 is well active in intestinal tissue during allo-HSCT (66, 100).

On the other hand, a tight relation between IL-22 and microbial flora has been observed. Thus, microbiota can directly induce IL-22 secretion by group 3 ILCs (25). Moreover, in epithelial tissues, IL-22 induces antimicrobial peptides, such as β-defensins 2 and 3, S100A8, and S100A9, which are able to specifically target certain microorganisms. An alteration in the composition of the microbiota was described in IL-22^−/−^ mice, and it largely increases the susceptibility to induced colitis (101). This particularity should be kept in mind when IL-22^−/−^ mice are used as recipients in gastrointestinal acute GVHD models. In addition to bacteria, the characterization of viral and fungal components of intestinal flora is in progress. It is probable that these components influence immune and also allogeneic responses. Recently, the role of the mycobiome has been addressed in different diseases (102, 103). Certain PAMPs, such as β-glucan or α-mannan polysaccharide chains that are part of the fungal membranes, can be, respectively, recognized by Dectin-1 and Dectin-2 receptors expressed by APC (104, 105). The recognition of certain patterns leads to Th17 responses and IL-17 and IL-22 production that control pathogens (106, 107). Thus, IL-22 can participate to the inflammation process in tissues in the context of fungal infection (108). In allo-HSCT, colonization by *Candida* is associated with an increase in GVHD severity (109). Interestingly, in patients with Dectin-1 polymorphism associated with a decreased Th17 response and an increase in *Candida* colonization, GVHD is not more severe (110). These data clearly suggest that the allogeneic response can be influenced by the mycobiome, but the relation between this and IL-22 and remains to be elucidated.

## Concluding Remarks

**Figure 3:**
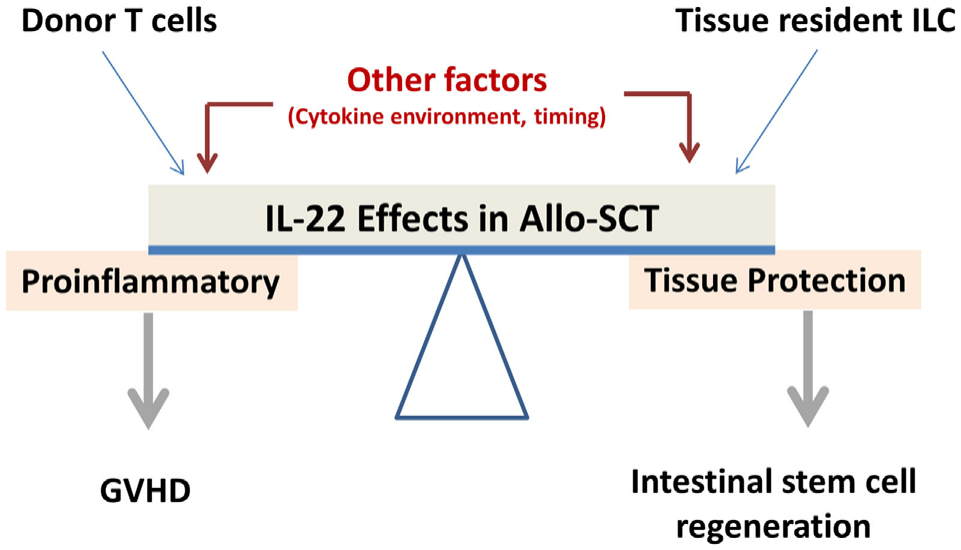
**Balance between inflammatory and protective effect of IL-22**.

A solid basis of data from *in vitro* and *in vivo* models is now accumulating to support IL-22s pleiotropic function. In particular, its dual pro-inflammatory and anti-inflammatory nature constitutes the largest obstacle to developing therapeutics based on this molecule (Figure 3). Therefore, further studies are required, especially in the human setting, to comprehensively explore the role of IL-22 in allo-HSCT.

## Author Contributions

BL wrote the manuscript and designed the figures, FM wrote the manuscript, PS and MM commented on the manuscript, and BG wrote the manuscript.

## Conflict of Interest Statement

The authors declare that the research was conducted in the absence of any commercial or financial relationships that could be construed as a potential conflict of interest.

## Footnotes

^1^http://www.businesswire.com/news/home/20120906005611/en/Generon-Initiates-Phase-Clinical-Study-F-652-Australia

^2^https://clinicaltrials.gov/ct2/show/NCT02406651?term=IL-22&rank=1

## References

[1] Ferrara JL Levine JE Reddy P Holler E. Graft-versus-host disease. Lancet (2009) 373(9674):1550–61. 10.1016/S0140-6736(09)60237-3 19282026 PMC2735047

[2] Markey KA MacDonald KP Hill GR. The biology of graft-versus-host disease: experimental systems instructing clinical practice. Blood (2014) 124(3):354–62. 10.1182/blood-2014-02-514745 24914137 PMC4102708

[3] Dumoutier L Louahed J Renauld JC. Cloning and characterization of IL-10-related T cell-derived inducible factor (IL-TIF), a novel cytokine structurally related to IL-10 and inducible by IL-9. J Immunol (2000) 164(4):1814–9. 10.4049/jimmunol.164.4.1814 10657629

[4] Xie MH Aggarwal S Ho WH Foster J Zhang Z Stinson J Interleukin (IL)-22, a novel human cytokine that signals through the interferon receptor-related proteins CRF2-4 and IL-22R. J Biol Chem (2000) 275(40):31335–9. 10.1074/jbc.M005304200 10875937

[5] Sabat R Wallace E Endesfelder S Wolk K. IL-19 and IL-20: two novel cytokines with importance in inflammatory diseases. Expert Opin Ther Targets (2007) 11(5):601–12. 10.1517/14728222.11.5.601 17465720

[6] Ouyang W Rutz S Crellin NK Valdez PA Hymowitz SG. Regulation and functions of the IL-10 family of cytokines in inflammation and disease. Annu Rev Immunol (2011) 29:71–109. 10.1146/annurev-immunol-031210-101312 21166540

[7] Logsdon NJ Jones BC Allman JC Izotova L Schwartz B Pestka S The IL-10R2 binding hot spot on IL-22 is located on the N-terminal helix and is dependent on N-linked glycosylation. J Mol Biol (2004) 342(2):503–14. 10.1016/j.jmb.2004.07.069 15327950

[8] Dumoutier L Van Roost E Colau D Renauld JC. Human interleukin-10-related T cell-derived inducible factor: molecular cloning and functional characterization as an hepatocyte-stimulating factor. Proc Natl Acad Sci U S A (2000) 97(18):10144–9. 10.1073/pnas.170291697 10954742 PMC27764

[9] Zindl CL Lai JF Lee YK Maynard CL Harbour SN Ouyang W IL-22-producing neutrophils contribute to antimicrobial defense and restitution of colonic epithelial integrity during colitis. Proc Natl Acad Sci U S A (2013) 110(31):12768–73. 10.1073/pnas.1300318110 23781104 PMC3732935

[10] Wolk K Kunz S Asadullah K Sabat R. Cutting edge: immune cells as sources and targets of the IL-10 family members? J Immunol (2002) 168(11):5397–402. 10.4049/jimmunol.168.11.5397 12023331

[11] Wolk K Kunz S Witte E Friedrich M Asadullah K Sabat R. IL-22 increases the innate immunity of tissues. Immunity (2004) 21(2):241–54. 10.1016/j.immuni.2004.07.007S1074761304001943 15308104

[12] Wolk K Witte E Wallace E Docke WD Kunz S Asadullah K IL-22 regulates the expression of genes responsible for antimicrobial defense, cellular differentiation, and mobility in keratinocytes: a potential role in psoriasis. Eur J Immunol (2006) 36(5):1309–23. 10.1002/eji.200535503 16619290

[13] Wolk K Witte K Witte E Proesch S Schulze-Tanzil G Nasilowska K Maturing dendritic cells are an important source of IL-29 and IL-20 that may cooperatively increase the innate immunity of keratinocytes. J Leukoc Biol (2008) 83(5):1181–93. 10.1189/jlb.0807525 18281438

[14] Duhen T Geiger R Jarrossay D Lanzavecchia A Sallusto F. Production of interleukin 22 but not interleukin 17 by a subset of human skin-homing memory T cells. Nat Immunol (2009) 10(8):857–63. 10.1038/ni.1767 19578369

[15] Trifari S Kaplan CD Tran EH Crellin NK Spits H. Identification of a human helper T cell population that has abundant production of interleukin 22 and is distinct from T(H)-17, T(H)1 and T(H)2 cells. Nat Immunol (2009) 10(8):864–71. 10.1038/ni.1770 19578368

[16] Ciric B El-behi M Cabrera R Zhang GX Rostami A. IL-23 drives pathogenic IL-17-producing CD8+ T cells. J Immunol (2009) 182(9):5296–305. 10.4049/jimmunol.0900036 19380776

[17] Hamada H Garcia-Hernandez Mde L Reome JB Misra SK Strutt TM McKinstry KK Tc17, a unique subset of CD8 T cells that can protect against lethal influenza challenge. J Immunol (2009) 182(6):3469–81. 10.4049/jimmunol.0801814 19265125 PMC2667713

[18] Liu Y Yang B Ma J Wang H Huang F Zhang J Interleukin-21 induces the differentiation of human Tc22 cells via phosphorylation of signal transducers and activators of transcription. Immunology (2011) 132(4):540–8. 10.1111/j.1365-2567.2010.03399.x 21214545 PMC3075507

[19] Martin B Hirota K Cua DJ Stockinger B Veldhoen M. Interleukin-17-producing gammadelta T cells selectively expand in response to pathogen products and environmental signals. Immunity (2009) 31(2):321–30. 10.1016/j.immuni.2009.06.020 19682928

[20] Monteleone G Pallone F Macdonald TT. Interleukin-21 (IL-21)-mediated pathways in T cell-mediated disease. Cytokine Growth Factor Rev (2009) 20(2):185–91. 10.1016/j.cytogfr.2009.02.002 19261537

[21] Sutton CE Lalor SJ Sweeney CM Brereton CF Lavelle EC Mills KH. Interleukin-1 and IL-23 induce innate IL-17 production from gammadelta T cells, amplifying Th17 responses and autoimmunity. Immunity (2009) 31(2):331–41. 10.1016/j.immuni.2009.08.001 19682929

[22] Ness-Schwickerath KJ Jin C Morita CT. Cytokine requirements for the differentiation and expansion of IL-17A- and IL-22-producing human Vgamma2Vdelta2 T cells. J Immunol (2010) 184(12):7268–80. 10.4049/jimmunol.1000600 20483730 PMC2965829

[23] Zheng Y Valdez PA Danilenko DM Hu Y Sa SM Gong Q Interleukin-22 mediates early host defense against attaching and effacing bacterial pathogens. Nat Med (2008) 14(3):282–9. 10.1038/nm1720 18264109

[24] Rutz S Eidenschenk C Ouyang W. IL-22, not simply a Th17 cytokine. Immunol Rev (2013) 252(1):116–32. 10.1111/imr.12027 23405899

[25] Satoh-Takayama N Vosshenrich CA Lesjean-Pottier S Sawa S Lochner M Rattis F Microbial flora drives interleukin 22 production in intestinal NKp46+ cells that provide innate mucosal immune defense. Immunity (2008) 29(6):958–70. 10.1016/j.immuni.2008.11.001 19084435

[26] Luci C Reynders A Ivanov II Cognet C Chiche L Chasson L Influence of the transcription factor RORgammat on the development of NKp46+ cell populations in gut and skin. Nat Immunol (2009) 10(1):75–82. 10.1038/ni.1681 19029904

[27] Sanos SL Bui VL Mortha A Oberle K Heners C Johner C RORgammat and commensal microflora are required for the differentiation of mucosal interleukin 22-producing NKp46+ cells. Nat Immunol (2009) 10(1):83–91. 10.1038/ni.1684 19029903 PMC4217274

[28] Takatori H Kanno Y Watford WT Tato CM Weiss G Ivanov II Lymphoid tissue inducer-like cells are an innate source of IL-17 and IL-22. J Exp Med (2009) 206(1):35–41. 10.1084/jem.20072713 19114665 PMC2626689

[29] Spits H Cupedo T. Innate lymphoid cells: emerging insights in development, lineage relationships, and function. Annu Rev Immunol (2012) 30:647–75. 10.1146/annurev-immunol-020711-075053 22224763

[30] Serafini N Klein Wolterink RG Satoh-Takayama N Xu W Vosshenrich CA Hendriks RW Gata3 drives development of RORgammat+ group 3 innate lymphoid cells. J Exp Med (2014) 211(2):199–208. 10.1084/jem.20131038 24419270 PMC3920560

[31] Aujla SJ Chan YR Zheng M Fei M Askew DJ Pociask DA IL-22 mediates mucosal host defense against Gram-negative bacterial pneumonia. Nat Med (2008) 14(3):275–81. 10.1038/nm1710 18264110 PMC2901867

[32] Andoh A Zhang Z Inatomi O Fujino S Deguchi Y Araki Y Interleukin-22, a member of the IL-10 subfamily, induces inflammatory responses in colonic subepithelial myofibroblasts. Gastroenterology (2005) 129(3):969–84. 10.1053/j.gastro.2005.06.071 16143135

[33] Sugimoto K Ogawa A Mizoguchi E Shimomura Y Andoh A Bhan AK IL-22 ameliorates intestinal inflammation in a mouse model of ulcerative colitis. J Clin Invest (2008) 118(2):534–44. 10.1172/JCI33194 18172556 PMC2157567

[34] Aggarwal S Xie MH Maruoka M Foster J Gurney AL. Acinar cells of the pancreas are a target of interleukin-22. J Interferon Cytokine Res (2001) 21(12):1047–53. 10.1089/107999001317205178 11798462

[35] Wolk K Witte E Hoffmann U Doecke WD Endesfelder S Asadullah K IL-22 induces lipopolysaccharide-binding protein in hepatocytes: a potential systemic role of IL-22 in Crohn’s disease. J Immunol (2007) 178(9):5973–81. 10.4049/jimmunol.178.9.5973 17442982

[36] Dudakov JA Hanash AM Jenq RR Young LF Ghosh A Singer NV Interleukin-22 drives endogenous thymic regeneration in mice. Science (2012) 336(6077):91–5. 10.1126/science.1218004 22383805 PMC3616391

[37] Dumoutier L Lejeune D Colau D Renauld JC. Cloning and characterization of IL-22 binding protein, a natural antagonist of IL-10-related T cell-derived inducible factor/IL-22. J Immunol (2001) 166(12):7090–5. 10.4049/jimmunol.166.12.7090 11390453

[38] Kotenko SV Izotova LS Mirochnitchenko OV Esterova E Dickensheets H Donnelly RP Identification, cloning, and characterization of a novel soluble receptor that binds IL-22 and neutralizes its activity. J Immunol (2001) 166(12):7096–103. 10.4049/jimmunol.166.12.7096 11390454

[39] Gruenberg BH Schoenemeyer A Weiss B Toschi L Kunz S Wolk K A novel, soluble homologue of the human IL-10 receptor with preferential expression in placenta. Genes Immun (2001) 2(6):329–34. 10.1038/sj.gene.6363786 11607789

[40] Weiss B Wolk K Grunberg BH Volk HD Sterry W Asadullah K Cloning of murine IL-22 receptor alpha 2 and comparison with its human counterpart. Genes Immun (2004) 5(5):330–6. 10.1038/sj.gene.6364104 15201862

[41] Xu W Presnell SR Parrish-Novak J Kindsvogel W Jaspers S Chen Z A soluble class II cytokine receptor, IL-22RA2, is a naturally occurring IL-22 antagonist. Proc Natl Acad Sci U S A (2001) 98(17):9511–6. 10.1073/pnas.171303198 11481447 PMC55483

[42] Huber S Gagliani N Zenewicz LA Huber FJ Bosurgi L Hu B IL-22BP is regulated by the inflammasome and modulates tumorigenesis in the intestine. Nature (2012) 491(7423):259–63. 10.1038/nature11535 23075849 PMC3493690

[43] Martin JC Beriou G Heslan M Chauvin C Utriainen L Aumeunier A Interleukin-22 binding protein (IL-22BP) is constitutively expressed by a subset of conventional dendritic cells and is strongly induced by retinoic acid. Mucosal Immunol (2013) 7(1):101–13. 10.1038/mi.2013.28 23653115 PMC4291114

[44] Martin JC Beriou G Heslan M Bossard C Jarry A Abidi A IL-22BP is produced by eosinophils in human gut and blocks IL-22 protective actions during colitis. Mucosal Immunol (2016) 9(2):539–49. 10.1038/mi.2015.83 26329427

[45] Boniface K Bernard FX Garcia M Gurney AL Lecron JC Morel F. IL-22 inhibits epidermal differentiation and induces proinflammatory gene expression and migration of human keratinocytes. J Immunol (2005) 174(6):3695–702. 10.4049/jimmunol.174.6.3695 15749908

[46] Brand S Beigel F Olszak T Zitzmann K Eichhorst ST Otte JM IL-22 is increased in active Crohn’s disease and promotes proinflammatory gene expression and intestinal epithelial cell migration. Am J Physiol Gastrointest Liver Physiol (2006) 290(4):G827–38. 10.1152/ajpgi.00513.2005 16537974

[47] Lejeune D Dumoutier L Constantinescu S Kruijer W Schuringa JJ Renauld JC. Interleukin-22 (IL-22) activates the JAK/STAT, ERK, JNK, and p38 MAP kinase pathways in a rat hepatoma cell line. Pathways that are shared with and distinct from IL-10. J Biol Chem (2002) 277(37):33676–82. 10.1074/jbc.M204204200 12087100

[48] Ikeuchi H Kuroiwa T Hiramatsu N Kaneko Y Hiromura K Ueki K Expression of interleukin-22 in rheumatoid arthritis: potential role as a proinflammatory cytokine. Arthritis Rheum (2005) 52(4):1037–46. 10.1002/art.20965 15818686

[49] Wang WB Levy DE Lee CK. STAT3 negatively regulates type I IFN-mediated antiviral response. J Immunol (2011) 187(5):2578–85. 10.4049/jimmunol.1004128 21810606

[50] Pickert G Neufert C Leppkes M Zheng Y Wittkopf N Warntjen M STAT3 links IL-22 signaling in intestinal epithelial cells to mucosal wound healing. J Exp Med (2009) 206(7):1465–72. 10.1084/jem.20082683 19564350 PMC2715097

[51] Zenewicz LA Yancopoulos GD Valenzuela DM Murphy AJ Stevens S Flavell RA. Innate and adaptive interleukin-22 protects mice from inflammatory bowel disease. Immunity (2008) 29(6):947–57. 10.1016/j.immuni.2008.11.003 19100701 PMC3269819

[52] Sonnenberg GF Fouser LA Artis D. Border patrol: regulation of immunity, inflammation and tissue homeostasis at barrier surfaces by IL-22. Nat Immunol (2011) 12(5):383–90. 10.1038/ni.2025 21502992

[53] Eyerich S Eyerich K Pennino D Carbone T Nasorri F Pallotta S Th22 cells represent a distinct human T cell subset involved in epidermal immunity and remodeling. J Clin Invest (2009) 119(12):3573–85. 10.1172/JCI40202 19920355 PMC2786807

[54] Wolk K Haugen HS Xu W Witte E Waggie K Anderson M IL-22 and IL-20 are key mediators of the epidermal alterations in psoriasis while IL-17 and IFN-gamma are not. J Mol Med (Berl) (2009) 87(5):523–36. 10.1007/s00109-009-0457-0 19330474

[55] Scheiermann P Bachmann M Goren I Zwissler B Pfeilschifter J Muhl H. Application of interleukin-22 mediates protection in experimental acetaminophen-induced acute liver injury. Am J Pathol (2013) 182(4):1107–13. 10.1016/j.ajpath.2012.12.010 23375450

[56] Muhl H. Pro-inflammatory signaling by IL-10 and IL-22: bad habit stirred up by interferons? Front Immunol (2013) 4:18. 10.3389/fimmu.2013.00018 23382730 PMC3562761

[57] Dumoutier L de Heusch M Orabona C Satoh-Takayama N Eberl G Sirard JC IL-22 is produced by gammaC-independent CD25+ CCR6+ innate murine spleen cells upon inflammatory stimuli and contributes to LPS-induced lethality. Eur J Immunol (2011) 41(4):1075–85. 10.1002/eji.201040878 21400499

[58] Savan R McFarland AP Reynolds DA Feigenbaum L Ramakrishnan K Karwan M A novel role for IL-22R1 as a driver of inflammation. Blood (2011) 117(2):575–84. 10.1182/blood-2010-05-285908 20971950 PMC3031481

[59] Leipe J Schramm MA Grunke M Baeuerle M Dechant C Nigg AP Interleukin 22 serum levels are associated with radiographic progression in rheumatoid arthritis. Ann Rheum Dis (2011) 70(8):1453–7. 10.1136/ard.2011.152074 21593004

[60] Geboes L Dumoutier L Kelchtermans H Schurgers E Mitera T Renauld JC Proinflammatory role of the Th17 cytokine interleukin-22 in collagen-induced arthritis in C57BL/6 mice. Arthritis Rheum (2009) 60(2):390–5. 10.1002/art.24220 19180498

[61] Boniface K Guignouard E Pedretti N Garcia M Delwail A Bernard FX A role for T cell-derived interleukin 22 in psoriatic skin inflammation. Clin Exp Immunol (2007) 150(3):407–15. 10.1111/j.1365-2249.2007.03511.x 17900301 PMC2219373

[62] Van Belle AB de Heusch M Lemaire MM Hendrickx E Warnier G Dunussi-Joannopoulos K IL-22 is required for imiquimod-induced psoriasiform skin inflammation in mice. J Immunol (2012) 188(1):462–9. 10.4049/jimmunol.1102224 22131335

[63] Nograles KE Zaba LC Guttman-Yassky E Fuentes-Duculan J Suarez-Farinas M Cardinale I Th17 cytokines interleukin (IL)-17 and IL-22 modulate distinct inflammatory and keratinocyte-response pathways. Br J Dermatol (2008) 159(5):1092–102. 10.1111/j.1365-2133.2008.08769.x 18684158 PMC2724264

[64] Sabat R Wolk K. Research in practice: IL-22 and IL-20: significance for epithelial homeostasis and psoriasis pathogenesis. J Dtsch Dermatol Ges (2011) 9(7):518–23. 10.1111/j.1610-0387.2011.07611.x 21251229

[65] Hanash AM Dudakov JA Hua G O’Connor MH Young LF Singer NV Interleukin-22 protects intestinal stem cells from immune-mediated tissue damage and regulates sensitivity to graft versus host disease. Immunity (2012) 37(2):339–50. 10.1016/j.immuni.2012.05.028 22921121 PMC3477611

[66] Couturier M Lamarthee B Arbez J Renauld JC Bossard C Malard F IL-22 deficiency in donor T cells attenuates murine acute graft-versus-host disease mortality while sparing the graft-versus-leukemia effect. Leukemia (2013) 27(7):1527–37. 10.1038/leu.2013.39 23399894

[67] Liang SC Nickerson-Nutter C Pittman DD Carrier Y Goodwin DG Shields KM IL-22 induces an acute-phase response. J Immunol (2010) 185(9):5531–8. 10.4049/jimmunol.0904091 20870942

[68] Zhao K Zhao D Huang D Song X Chen C Pan B The identification and characteristics of IL-22-producing T cells in acute graft-versus-host disease following allogeneic bone marrow transplantation. Immunobiology (2013) 218(12):1505–13. 10.1016/j.imbio.2013.05.005 23816304

[69] Zhao K Zhao D Huang D Yin L Chen C Pan B Interleukin-22 aggravates murine acute graft-versus-host disease by expanding effector T cell and reducing regulatory T cell. J Interferon Cytokine Res (2014) 34(9):707–15. 10.1089/jir.2013.0099 24720737

[70] Edinger M Hoffmann P. Regulatory T cells in stem cell transplantation: strategies and first clinical experiences. Curr Opin Immunol (2011) 23(5):679–84. 10.1016/j.coi.2011.06.006 21802270

[71] Ganguly S Ross DB Panoskaltsis-Mortari A Kanakry CG Blazar BR Levy RB Donor CD4+ Foxp3+ regulatory T cells are necessary for post-transplantation cyclophosphamide-mediated protection against GVHD in mice. Blood (2014) 124(13):2131–41. 10.1182/blood-2013-10-525873 25139358 PMC4186542

[72] Okamoto S Fujiwara H Nishimori H Matsuoka K Fujii N Kondo E Anti-IL-12/23 p40 antibody attenuates experimental chronic graft-versus-host disease via suppression of IFN-gamma/IL-17-producing cells. J Immunol (2015) 194(3):1357–63. 10.4049/jimmunol.1400973 25527789

[73] Brüggen MC Klein I Greinix H Bauer W Kuzmina Z Rabitsch W Diverse T-cell responses characterize the different manifestations of cutaneous graft-versus-host disease. Blood (2014) 123(2):290–9. 10.1182/blood-2013-07-514372 24255916

[74] Tumino M Serafin V Accordi B Spadini S Forest C Cortese G Interleukin-22 in the diagnosis of active chronic graft-versus-host disease in paediatric patients. Br J Haematol (2015) 168(1):142–5. 10.1111/bjh.13068 25098229

[75] Lamarthee B Malard F Gamonet C Bossard C Couturier M Renauld JC Donor interleukin-22 and host type I interferon signaling pathway participate in intestinal graft-versus-host disease via STAT1 activation and CXCL10. Mucosal Immunol (2016) 9(2):309–21. 10.1038/mi.2015.61 26153763

[76] Takaoka A Mitani Y Suemori H Sato M Yokochi T Noguchi S Cross talk between interferon-gamma and -alpha/beta signaling components in caveolar membrane domains. Science (2000) 288(5475):2357–60. 10.1126/science.288.5475.2357 10875919

[77] Mitani Y Takaoka A Kim SH Kato Y Yokochi T Tanaka N Cross talk of the interferon-alpha/beta signalling complex with gp130 for effective interleukin-6 signalling. Genes Cells (2001) 6(7):631–40. 10.1046/j.1365-2443.2001.00448.x 11473581

[78] Hoegl S Bachmann M Scheiermann P Goren I Hofstetter C Pfeilschifter J Protective properties of inhaled IL-22 in a model of ventilator-induced lung injury. Am J Respir Cell Mol Biol (2011) 44(3):369–76. 10.1165/rcmb.2009-0440OC 20463292

[79] Song MM Shuai K. The suppressor of cytokine signaling (SOCS) 1 and SOCS3 but not SOCS2 proteins inhibit interferon-mediated antiviral and antiproliferative activities. J Biol Chem (1998) 273(52):35056–62. 10.1074/jbc.273.52.35056 9857039

[80] Lee TH Lee JY Park S Shin SH Yahng SA Yoon JH Expression of SOCS1 and SOCS3 genes in human graft-versus-host disease after allogeneic hematopoietic stem cell transplantation. Blood Res (2013) 48(1):16–23. 10.5045/br.2013.48.1.16 23589790 PMC3625005

[81] Leng C Gries M Ziegler J Lokshin A Mascagni P Lentzsch S Reduction of graft-versus-host disease by histone deacetylase inhibitor suberonylanilide hydroxamic acid is associated with modulation of inflammatory cytokine milieu and involves inhibition of STAT1. Exp Hematol (2006) 34(6):776–87. 10.1016/j.exphem.2006.02.014 16728283

[82] Imanguli MM Swaim WD League SC Gress RE Pavletic SZ Hakim FT. Increased T-bet+ cytotoxic effectors and type I interferon-mediated processes in chronic graft-versus-host disease of the oral mucosa. Blood (2009) 113(15):3620–30. 10.1182/blood-2008-07-168351 19168793 PMC2668847

[83] Ihle JN Kerr IM. Jaks and Stats in signaling by the cytokine receptor superfamily. Trends Genet (1995) 11(2):69–74. 10.1016/S0168-9525(00)89000-9 7716810

[84] Cetkovic-Cvrlje M Roers BA Schonhoff D Waurzyniak B Liu XP Uckun FM. Treatment of post-bone marrow transplant acute graft-versus-host disease with a rationally designed JAK3 inhibitor. Leuk Lymphoma (2002) 43(7):1447–53. 10.1080/1042819022386581 12389628

[85] Betts BC Abdel-Wahab O Curran SA St Angelo ET Koppikar P Heller G Janus kinase-2 inhibition induces durable tolerance to alloantigen by human dendritic cell-stimulated T cells yet preserves immunity to recall antigen. Blood (2011) 118(19):5330–9. 10.1182/blood-2011-06-363408 21917753 PMC3217413

[86] Spoerl S Mathew NR Bscheider M Schmitt-Graeff A Chen S Mueller T Activity of therapeutic JAK 1/2 blockade in graft-versus-host disease. Blood (2014) 123(24):3832–42. 10.1182/blood-2013-12-543736 24711661

[87] Capitini CM Nasholm NM Chien CD Larabee SM Qin H Song YK Absence of STAT1 in donor-derived plasmacytoid dendritic cells results in increased STAT3 and attenuates murine GVHD. Blood (2014) 124(12):1976–86. 10.1182/blood-2013-05-500876 25079358 PMC4168352

[88] Zohar Y Wildbaum G Novak R Salzman AL Thelen M Alon R CXCL11-dependent induction of FOXP3-negative regulatory T cells suppresses autoimmune encephalomyelitis. J Clin Invest (2014) 124(5):2009–22. 10.1172/JCI71951 24713654 PMC4001543

[89] Bossard C Malard F Arbez J Chevallier P Guillaume T Delaunay J Plasmacytoid dendritic cells and Th17 immune response contribution in gastrointestinal acute graft-versus-host disease. Leukemia (2012) 26(7):1471–4. 10.1038/leu.2012.41 22333879

[90] Malard F Bossard C Brissot E Chevallier P Guillaume T Delaunay J Increased plasmacytoid dendritic cells and RORgammat-expressing immune effectors in cutaneous acute graft-versus-host disease. J Leukoc Biol (2013) 94(6):1337–43. 10.1189/jlb.0513295 23990625

[91] Tohyama M Yang L Hanakawa Y Dai X Shirakata Y Sayama K IFN-alpha enhances IL-22 receptor expression in keratinocytes: a possible role in the development of psoriasis. J Invest Dermatol (2012) 132(7):1933–5. 10.1038/jid.2011.468 22297633

[92] Munneke JM Bjorklund AT Mjosberg JM Garming-Legert K Bernink JH Blom B Activated innate lymphoid cells are associated with a reduced susceptibility to graft versus host disease. Blood (2014) 124(5):812–21. 10.1182/blood-2013-11-536888 24855210

[93] Lindemans CA Calafiore M Mertelsmann AM O’Connor MH Dudakov JA Jenq RR Interleukin-22 promotes intestinal-stem-cell-mediated epithelial regeneration. Nature (2015) 528(7583):560–4. 10.1038/nature16460 26649819 PMC4720437

[94] Huber S Gagliani N Flavell RA. Life, death, and miracles: Th17 cells in the intestine. Eur J Immunol (2012) 42(9):2238–45. 10.1002/eji.201242619 22949322

[95] Schroder K Tschopp J. The inflammasomes. Cell (2010) 140(6):821–32. 10.1016/j.cell.2010.01.040 20303873

[96] Reddy P Ferrara JL. Role of interleukin-18 in acute graft-vs-host disease. J Lab Clin Med (2003) 141(6):365–71. 10.1016/S0022-2143(03)00028-3 12819633

[97] Scholl S Sayer HG Mugge LO Kasper C Pietraszczyk M Kliche KO Increase of interleukin-18 serum levels after engraftment correlates with acute graft-versus-host disease in allogeneic peripheral blood stem cell transplantation. J Cancer Res Clin Oncol (2004) 130(12):704–10. 10.1007/s00432-004-0603-6 15365821 PMC12161803

[98] Munoz M Eidenschenk C Ota N Wong K Lohmann U Kuhl AA Interleukin-22 induces interleukin-18 expression from epithelial cells during intestinal infection. Immunity (2015) 42(2):321–31. 10.1016/j.immuni.2015.01.011 25680273

[99] Ferrara JL Harris AC Greenson JK Braun TM Holler E Teshima T Regenerating islet-derived 3-alpha is a biomarker of gastrointestinal graft-versus-host disease. Blood (2011) 118(25):6702–8. 10.1182/blood-2011-08-375006 21979939 PMC3242723

[100] Eriguchi Y Uryu H Nakamura K Shimoji S Takashima S Iwasaki H Reciprocal expression of enteric antimicrobial proteins in intestinal graft-versus-host disease. Biol Blood Marrow Transplant (2013) 19(10):1525–9. 10.1016/j.bbmt.2013.07.027 23927965

[101] Zenewicz LA Yin X Wang G Elinav E Hao L Zhao L IL-22 deficiency alters colonic microbiota to be transmissible and colitogenic. J Immunol (2013) 190(10):5306–12. 10.4049/jimmunol.1300016 23585682 PMC3646987

[102] Huffnagle GB Noverr MC. The emerging world of the fungal microbiome. Trends Microbiol (2013) 21(7):334–41. 10.1016/j.tim.2013.04.002 23685069 PMC3708484

[103] Iliev ID Funari VA Taylor KD Nguyen Q Reyes CN Strom SP Interactions between commensal fungi and the C-type lectin receptor Dectin-1 influence colitis. Science (2012) 336(6086):1314–7. 10.1126/science.1221789 22674328 PMC3432565

[104] Brown GD Taylor PR Reid DM Willment JA Williams DL Martinez-Pomares L Dectin-1 is a major beta-glucan receptor on macrophages. J Exp Med (2002) 196(3):407–12. 10.1084/jem.20020470 12163569 PMC2193936

[105] Netea MG Brown GD Kullberg BJ Gow NA. An integrated model of the recognition of *Candida albicans* by the innate immune system. Nat Rev Microbiol (2008) 6(1):67–78. 10.1038/nrmicro1815 18079743

[106] Gessner MA Werner JL Lilly LM Nelson MP Metz AE Dunaway CW Dectin-1-dependent interleukin-22 contributes to early innate lung defense against *Aspergillus fumigatus*. Infect Immun (2012) 80(1):410–7. 10.1128/IAI.05939-11 22038916 PMC3255669

[107] Saijo S Ikeda S Yamabe K Kakuta S Ishigame H Akitsu A Dectin-2 recognition of alpha-mannans and induction of Th17 cell differentiation is essential for host defense against *Candida albicans*. Immunity (2010) 32(5):681–91. 10.1016/j.immuni.2010.05.001 20493731

[108] Lilly LM Gessner MA Dunaway CW Metz AE Schwiebert L Weaver CT The beta-glucan receptor dectin-1 promotes lung immunopathology during fungal allergy via IL-22. J Immunol (2012) 189(7):3653–60. 10.4049/jimmunol.1201797 22933634 PMC3448838

[109] Marr KA Seidel K Slavin MA Bowden RA Schoch HG Flowers ME Prolonged fluconazole prophylaxis is associated with persistent protection against candidiasis-related death in allogeneic marrow transplant recipients: long-term follow-up of a randomized, placebo-controlled trial. Blood (2000) 96(6):2055–61. 10979947

[110] van der Velden WJ Plantinga TS Feuth T Donnelly JP Netea MG Blijlevens NM. The incidence of acute graft-versus-host disease increases with *Candida* colonization depending the dectin-1 gene status. Clin Immunol (2010) 136(2):302–6. 10.1016/j.clim.2010.04.007 20452827

